# Smart Formulation: AI-Driven Web Platform for Optimization and Stability Prediction of Compounded Pharmaceuticals Using KNIME

**DOI:** 10.3390/ph18081240

**Published:** 2025-08-21

**Authors:** Artur Grigoryan, Stefan Helfrich, Valentin Lequeux, Benjamine Lapras, Chloé Marchand, Camille Merienne, Fabien Bruno, Roseline Mazet, Fabrice Pirot

**Affiliations:** 1 Fripharm^®^, Pharmacy Department, Groupe Hospitalier Centre Edouard Herriot, Hospices Civils de Lyon, 5, Place d′Arsonval, F-69437 Lyon, France; artur.grigoryan@etu.univ-lyon1.fr (A.G.); valentin.lequeux@chu-lyon.fr (V.L.); benjamine.lapras@chu-lyon.fr (B.L.); chloe.marchand@chu-lyon.fr (C.M.); camille.merienne@chu-lyon.fr (C.M.); 2 KNIME GmbH, Reichenaustr. 11, DE-78467 Konstanz, Germany; stefan.helfrich@knime.com; 3 Pharmacie Delpech, 5 rue Danton, F-75006 Paris, France; fabienbruno@delpechparis.com; 4 Pharmacy Department, CHU Grenoble Alpes, 38000 Grenoble, France; rmazet@chu-grenoble.fr; 5 Laboratoire de Pharmacie Galénique Industrielle, UMR 5305, Plateforme Fripharm, ISPB-Faculté de Pharmacie, Université Claude Bernard Lyon 1, 8, Avenue Rockefeller, F-69373 Lyon, France

**Keywords:** pharmaceutical stability, beyond-use date prediction, machine learning, molecular descriptors, drug compounding, excipients

## Abstract

**Background/Objectives**: *Smart Formulation* is an artificial intelligence-based platform designed to predict the Beyond Use Dates (BUDs) of compounded oral solid dosage forms. The study aims to develop a decision-support tool for pharmacists by integrating molecular, formulation, and environmental parameters to assist in optimizing the stability of extemporaneous preparations. **Methods**: A tree ensemble regression model was trained using a curated dataset of 55 experimental BUD values collected from the Stabilis database. Each formulation was encoded with molecular descriptors, excipient composition, packaging type, and storage conditions. The model was implemented using the KNIME platform, allowing the integration of cheminformatics and machine learning workflows. After training, the model was used to predict BUDs for 3166 APIs under various formulation and storage scenarios. **Results**: The analysis revealed a significant impact of excipient type, number, and environmental conditions on API stability. APIs with lower LogP values generally exhibited greater stability, particularly when formulated with a single excipient. Excipients such as cellulose, silica, sucrose, and mannitol were associated with improved stability, whereas HPMC and lactose contributed to faster degradation. The use of two excipients instead of one frequently resulted in reduced BUDs, possibly due to moisture redistribution or phase separation effects. **Conclusions**: *Smart Formulation* represents a valuable contribution to computational pharmaceutics, bridging theoretical formulation design with practical compounding needs. The platform offers a scalable, cost-effective alternative to traditional stability testing and is already available for use by healthcare professionals. Its implementation in hospital and community pharmacies may help mitigate drug shortages, support formulation standardization, and improve patient care. Future developments will focus on real-time stability monitoring and adaptive learning for enhanced precision.

## 1. Introduction

The supply of medicines poses a significant challenge for healthcare systems globally, exacerbated by the increasing shortages of essential medicines [1,2]. In response to these challenges, hospital and community pharmacies play a vital role in ensuring continuity of care, often resorting to pharmaceutical compounding to mitigate the absence of certain licensed medicines [3,4].

While compounding offers a critical solution to drug shortages, formulation errors, contamination, and variability in quality may undermine patient safety and treatment efficacy. Studies have reported cases of compounding errors leading to adverse health outcomes, underscoring the need for stringent quality control measures [5,6]. Regulatory oversight is crucial to ensuring that compounded medications meet appropriate safety and efficacy standards, distinguishing pharmaceutical compounding from large-scale drug manufacturing [7]. The European Drug Shortages Formulary Project emphasizes this need by establishing a regulatory framework for the use of compounded preparations in shortage management [2].

In this context, the development of a formulation algorithm dedicated to pharmaceutical compounding of oral solid dosage form would represent a significant advance in optimizing the quality and safety of unlicensed preparations. By applying Quality by Design principles to pharmaceutical compounding, it becomes possible to pre-evaluate and improve the stability of formulations while minimizing the risks associated with those preparations [7,8]. Such a tool would also facilitate the rapid adjustment of formulations to meet specific clinical needs and address fluctuations in raw material availability [9], bypassing the lengthy stability studies required by the International Council for Harmonisation (ICH) guidelines. Moreover, these long-term studies, which can take months to complete, are impractical in time-sensitive situations requiring immediate therapeutic solutions [10,11].

However, implementing such an algorithm requires strict adherence to existing standards and regulations, including those outlined in USP-NF 795 and 797 for non-sterile and sterile preparations [12,13] as well as the European Pharmacopoeia [14]. By structuring data from the scientific literature and pharmaceutical practices in both community and hospital settings, this algorithm could offer an innovative and effective solution to mitigate the impact of drug shortages while ensuring the quality and safety of unlicensed preparations.

Artificial intelligence (AI) has emerged as a transformative tool in pharmaceutical sciences, driving innovations in drug discovery [15], formulation development [16], drug delivery [17,18], pharmaceutical dosage form testing [19], and personalized medicine [16]. More specifically, AI technologies have demonstrated their utility in developing solid dosage forms [20,21], predicting solid dispersion stability [22] and evaluating drug-excipient compatibility (Wang, 2021) [18]. AI-based models allow for the prediction and optimization of pharmaceutical formulations by leveraging large datasets from experimental stability studies and computational simulations. Like AI-driven platforms such as *PharmSD*, *Smart Formulation* employs machine learning techniques to identify the most stable active pharmaceutical ingredient (API)-excipient combinations, minimizing formulation failures and reducing the need for a number of extensive experimental trials. Furthermore, *Smart Formulation* enables the precise estimation of beyond use dates (BUDs) under different storage conditions, thus enhancing patient safety and the reliability of unlicensed preparations.

Building on these advancements, *Smart Formulation* emerges as an AI-powered expert system designed to optimize pharmaceutical compounding by integrating over 3000 APIs, which were extracted from *ChEMBL* and *PubChem* web services [23,24]. The system also incorporates a selection of six commonly used excipients and three types of packaging materials (glass, plastic, and paper) [25,26,27]. The modeling protocol, which includes the curation of chemical and experimental data, was implemented in KNIME to automate the stability prediction workflow [28,29,30,31]. Specifically, 53 stability data points of oral solid preparations were extracted from the *Stabilis* database [32] to form a training dataset, which was then used to develop a machine learning model within KNIME. The model’s accuracy and relevance were evaluated by analyzing the correlation between predicted and experimental BUD values. Furthermore, the impact of API chemical properties in combination with one or two excipients on BUD prediction was assessed using the training dataset. To validate the approach, a portion of the *Smart Formulation* predictions was compared to those generated by the AI-driven *FormulationAI* platform [33]. Additionally, the predicted BUDs of 27 extemporaneous oral solid preparations were compared with against the reference values provided by official unlicensed preparation formularies, including the Belgian magistral therapeutic formulary (FTM) [34], the Dutch Formularium der Nederlandse Apothekers [35], the German Deutscher Arzneimittel-Codex/Neues Rezeptur-Formularium [36], and the National Formulary of the French Pharmacopoeia [37]. Finally, the BUDs of preparations reported in the scientific literature and pharmaceutical formularies will be compared to (i) those predicted by *Smart Formulation* and *FormulationAI*, (ii) the declared shelf-life of raw materials provided by manufacturers, and (iii) the expiration dates of commercially available pharmaceutical products. This comparative analysis will assess the robustness and relevance of the *Smart Formulation* predictive model.

## 2. Results and Discussion

Solid oral dosage forms continue to play a pivotal role in pharmaceutical development, offering advantages such as chemical and physical stability, ease of storage and transport, high patient acceptability, and cost-efficient mass production. Conventional manufacturing techniques such as direct compression, wet or dry granulation, and capsule filling remain well-established for producing robust, standardized formulations. Nonetheless, recent advances **in three-dimensional printing** have introduced transformative possibilities for **personalized medicine**, enabling the on-demand production of dosage forms with customized release profiles, complex geometries, and precise drug loading [38]. The convergence of traditional manufacturing and additive technologies creates a unique framework for developing both **scalable and patient-specific** therapies.

### 2.1. AMF-DB and Smart Formulation Development and Model Evaluation

**Figure 1:**
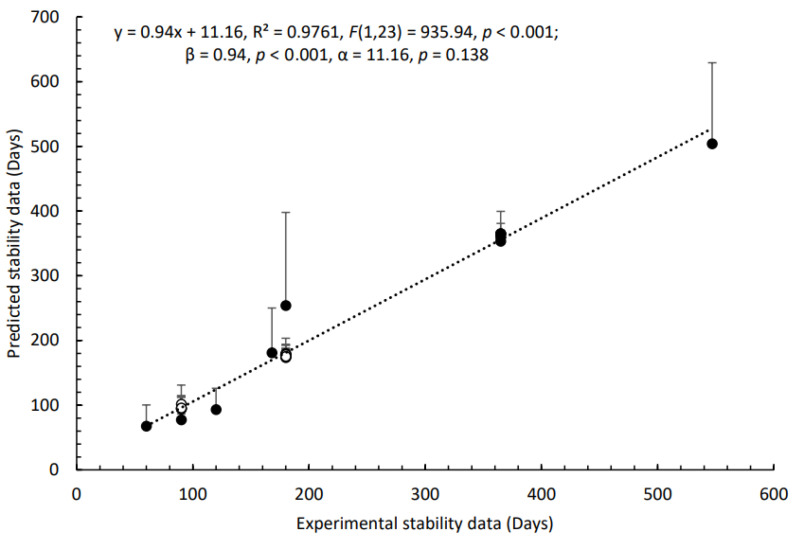
The relationship between predicted and experimental stability data was significantly estimated by the Tree Ensemble Regression learner and predictor-based model. (◯) excipients (pure or blended with one other excipient, n = 14 data points), (●) API (compounded with one or two excipients, n = 11 data points) conditioned in glass, plastic, and paper packaging (cf. *0 Stability Predictor Model* panel in Figure 1). API content and temperature storage were between 0.2 and 100%, and 4 °C, 25 °C, and 40 °C, respectively. (n = 14) demonstrated consistent predictability across glass, plastic, and paper packaging. A comparative analysis of seven machine learning models was conducted to evaluate predictive performance (Table 1). Among the tested models, Tree Ensemble Regression exhibited the highest accuracy (R^2^ = 0.975) with the lowest Root Mean Squared Error (RMSE = 18.93) and Mean Absolute Error (MAE = 10.16). In contrast, other ensemble and boosting methods showed varying degrees of performance, with Gradient Boosted Trees Regression yielding suboptimal results (R^2^ = -0.447, RMSE = 137.60). These findings validate the robustness of *Smart Formulation* in predicting drug stability under diverse formulation and storage conditions. The model effectively captures key interactions between API content, excipients, packaging, and temperature, making it a reliable tool for pharmaceutical stability assessment.

To assess the stability of pharmaceutical formulations, we developed the *Smart Formulation* predictive model using the AMF-DB database. AMF-DB integrates molecular descriptors, formulation parameters (including excipients and packaging types), and storage conditions to estimate BUDs. The predictive workflow was implemented in KNIME, leveraging machine learning techniques to enhance accuracy. The predictive accuracy of *Smart Formulation* was assessed by comparing the predicted stability data with experimental values. Figure 1 illustrates the correlation between the predicted and experimental stability data, showing a strong linear relationship (R^2^ = 0.9761, *p* < 0.001). The model, based on a Tree Ensemble Regression approach, effectively predicted stability across different formulation conditions, notably, APIs formulated with one or two excipients (n = 11) and those in pure or binary excipient mixtures.

**Table 1:** Comparative performance of seven KNIME models for drug stability prediction: analysis of drug content, storage conditions, packaging, and experimental data using single decision, ensemble, and boosting methods.

Metrics	Single Decision Method	Ensemble Methods				Boosting Methods	
	Decision Trees	Random Forests	Random Forest Regression	Tree Ensembles	*Tree Ensembles* *Regression*	Gradient Boosted Trees	Gradient Boosted Trees Regression
R^2^	0.93	0.758	0.536	0.491	***0.975***	−0.447	0.849
Mean Absolute Error (MAE)	9	17.94	48.86	24.54	***10.16***	98.17	19.45
Mean Squared Error (MSE)	912.90	3160.86	6073.61	6661.17	***358.16***	18,931.90	1972.69
Root Mean Squared Error (RMSE)	30.21	56.22	77.93	81.62	***18.93***	137.60	44.42
Mean Signed Difference (MSD)	−1.17	8.34	20.20	24.54	***−0.13***	−76.46	−7.15
Mean Absolute Percentage Error (MAPE)	0.05	0.23	0.59	0.44	***0.064***	0.50	0.16

### 2.2. Model Validation and Performance Testing

To further evaluate the predictive capabilities of *Smart Formulation*, a correlation analysis was conducted to determine the influence of molecular descriptors, formulation parameters, packaging, and storage conditions on the predicted BUDs (Table 2). The analysis revealed that LogP (R = 0.503, *p* = 0.012) and LogP class (R = 0.502, *p* = 0.012) were significantly correlated with BUD predictions, suggesting that lipophilicity plays a key role in drug stability under various conditions. Additionally, the molecule class exhibited a moderate correlation (R = 0.439, *p* = 0.032), indicating that structural characteristics of APIs contribute to their degradation profile. In contrast, most formulation and storage parameters showed weaker correlations, with the main excipient (R = 0.041, *p* = 0.849) and packaging type (R = −0.200, *p* = 0.349) not significantly influencing BUD predictions. While temperature demonstrated a negative trend (R = −0.336, *p* = 0.109), it did not reach statistical significance, suggesting that its effect might be better captured through interaction terms or non-linear modeling approaches. These findings highlight the dominant role of molecular descriptors, particularly lipophilicity and structural classification, in predicting the stability of compounded APIs. These statistical correlations should be interpreted with caution, especially given the limited dataset. The *Smart Formulation* platform is designed primarily as a decision-support tool to assist pharmacists in identifying relevant formulation factors, rather than as a source of definitive predictions.

The prediction of beyond use dates (BUDs) for compounded pharmaceutical products is a highly complex task. It involves multiple interdependent variables such as degradation kinetics (which are often non-linear), the physicochemical properties of the API, crystallinity, excipient interactions, packaging, and environmental factors like temperature and humidity. To account for this complexity, we included a wide set of molecular descriptors and formulation-related variables in the model, aiming to capture as many indirect indicators as possible within the limits of the available data. While we acknowledge that such complexity cannot be fully captured with a small dataset, our approach demonstrates the feasibility of applying machine learning in this domain and sets the foundation for future development as more comprehensive stability data become accessible.

**Figure 2:**
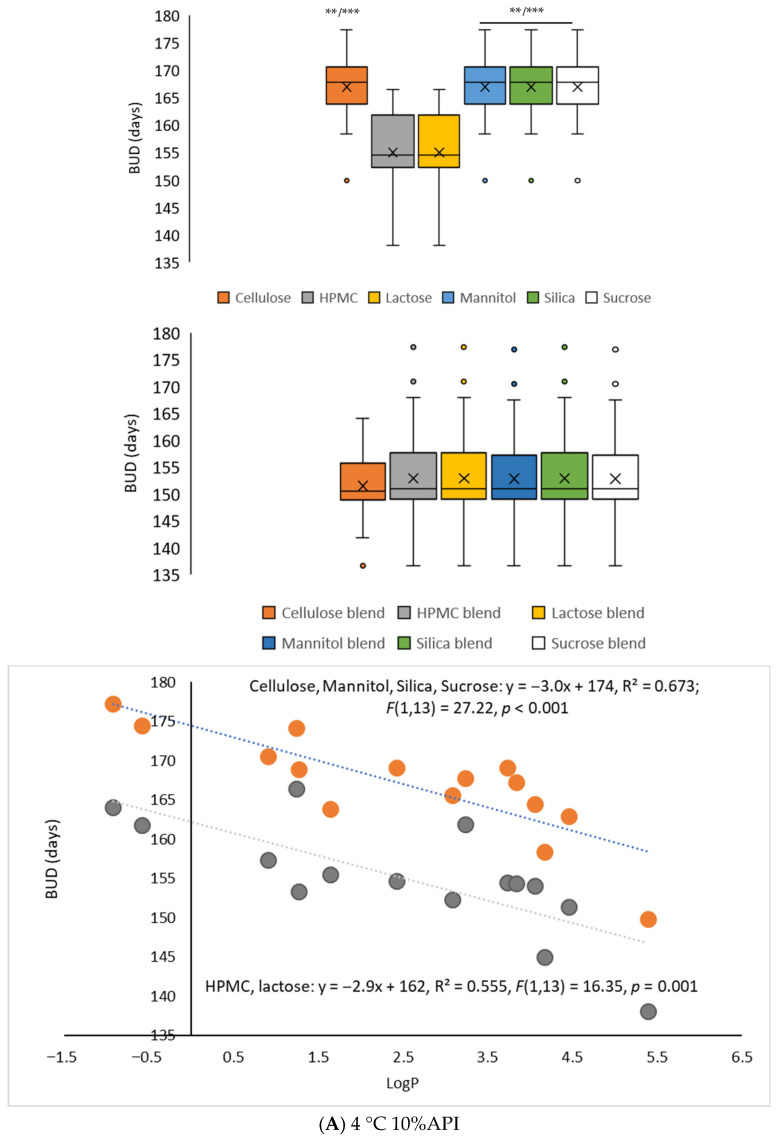

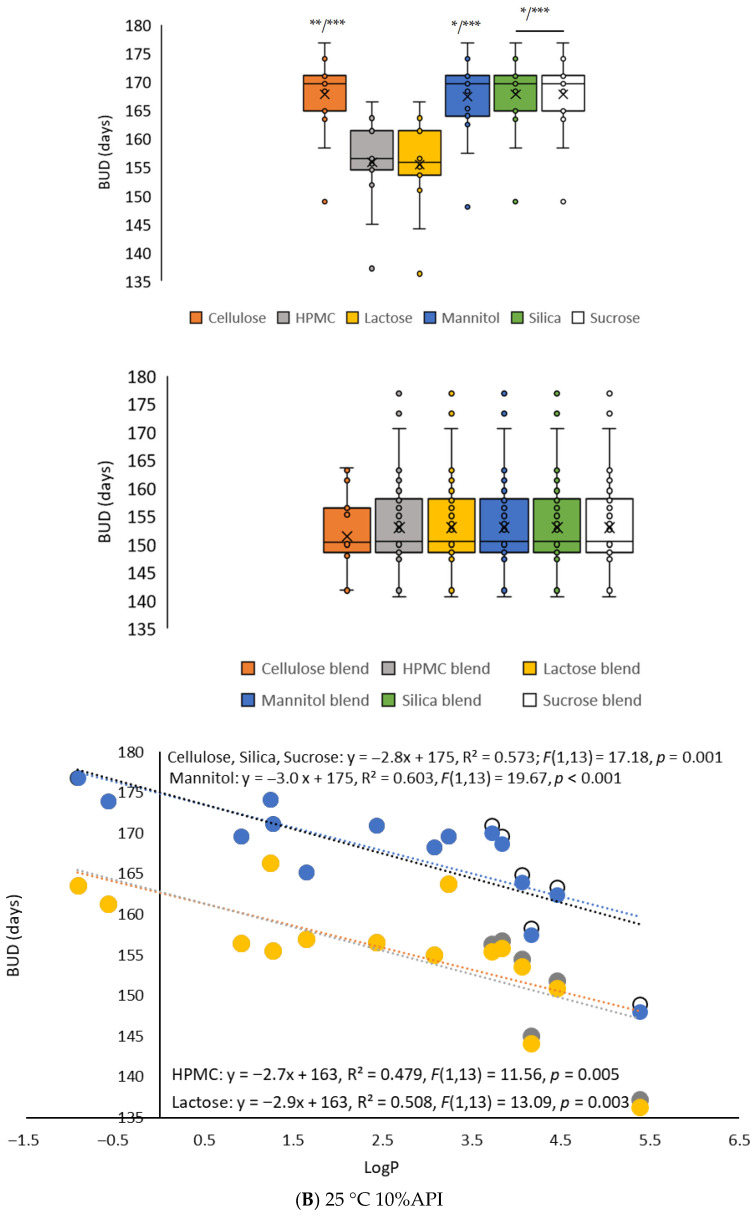

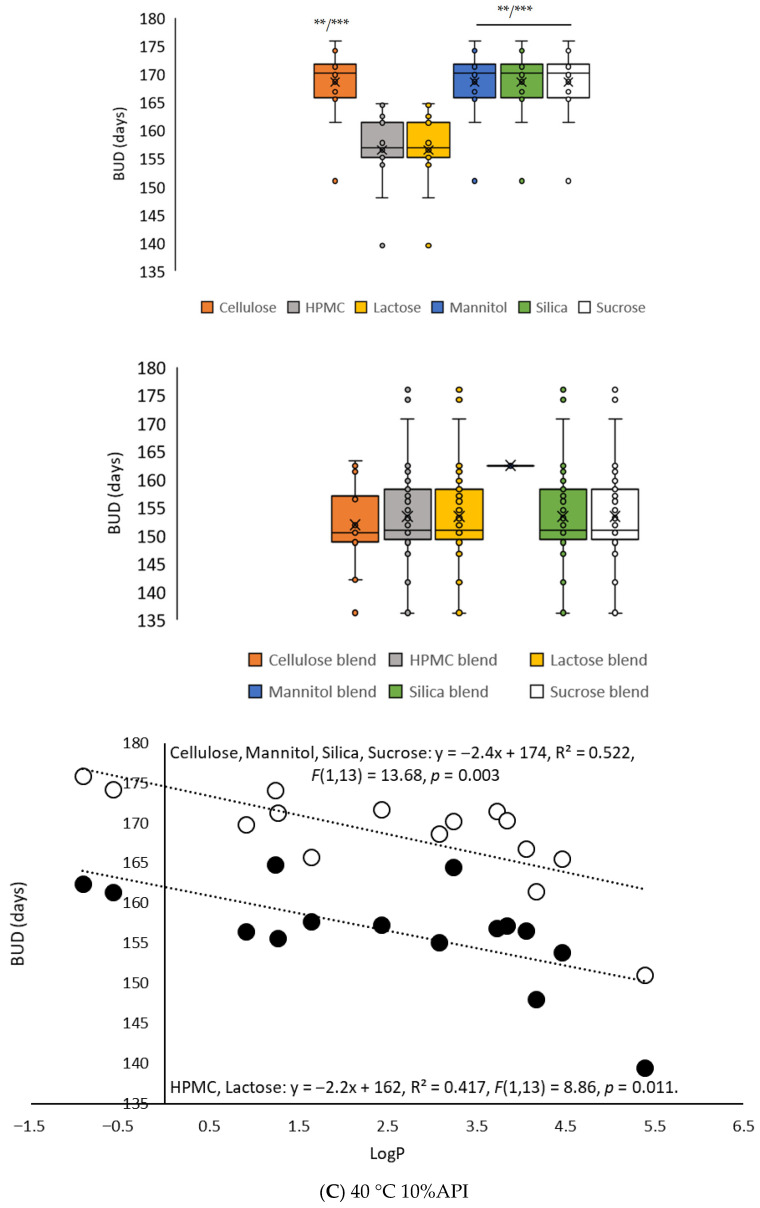

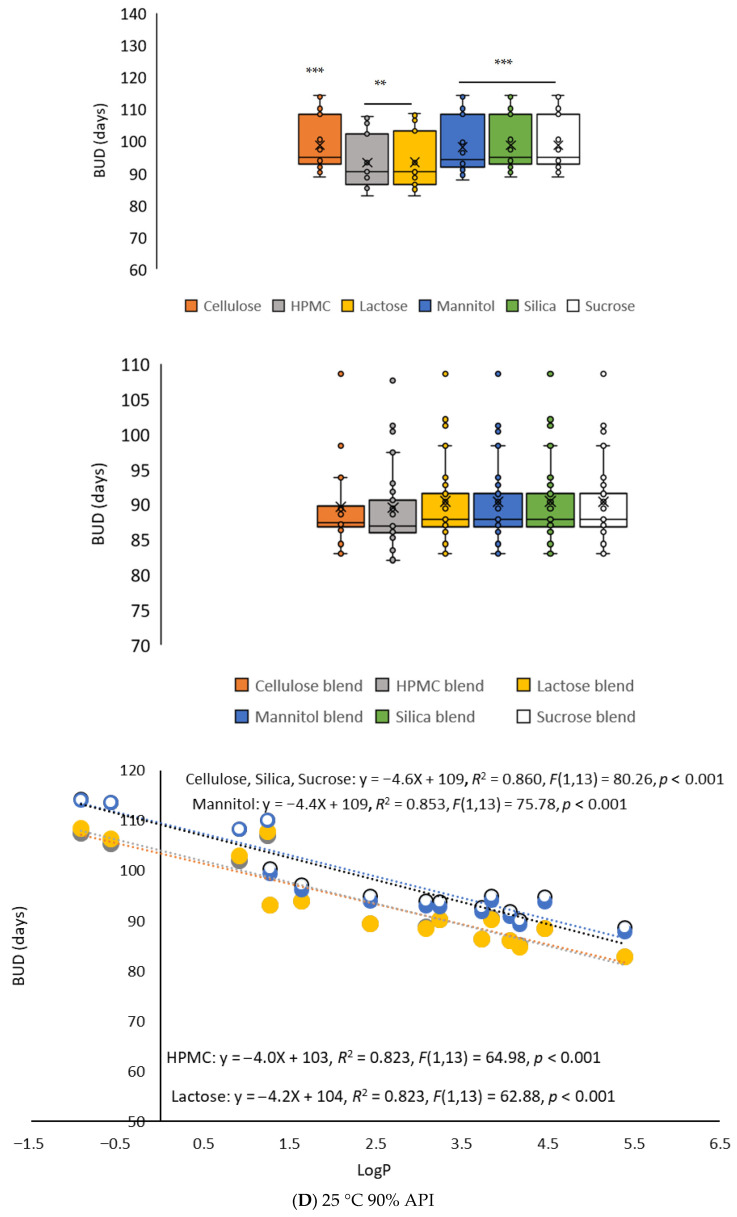
Prediction of API (n = 15; MW: 129.17 to 776.87 g.mol^−1^, LogP: −0.92 to 5.39; initial content: 10%, conditioning: plastic) stability in 6 pure or 15 combinations of blended excipients as a function of temperature of storage. (**A**): 4 °C storage, API content: 10%, **: *p* < 0.01 as compared to HPMC and Lactose groups; ***: *p* < 0.0001 as compared to blend groups (ANOVA, post hoc Tukey’s All pairs comparison. A significant linear relationship between prediction stability and LogP of API is shown in presence of either (●) cellulose, mannitol, silica, and sucrose pure excipients, or (●) HPMC and mannitol pure excipients. Shapiro–Wilk *p*-value > 0.05. (**B**): 25 °C storage, API content: 10%, *p < 0.05 and **: *p* < 0.01 as compared to HPMC and Lactose groups; ***: *p* < 0.0001 as compared to blend groups. A significant linear relationship between prediction stability and LogP of API is shown in presence of either (◯) cellulose, silica and sucrose, (●) mannitol, (●) HPMC or (●) lactose pure excipients. Shapiro–Wilk *p*-value > 0.05. (**C**): 40 °C storage, API content: 10%, a, **: *p* < 0.001 as compared to HPMC and Lactose groups; b, ***: *p* < 0.0001 as compared to cellulose, HPMC, lactose, silica, and sucrose blend groups. A significant linear relationship between prediction stability and LogP of API is shown in presence of either (◯) cellulose, mannitol, silica, and sucrose pure excipients, or (●) HPMC and mannitol pure excipients. Shapiro–Wilk *p*-value > 0.05. (**D**): 25 °C storage, API content: 90%, **: *p* < 0.01 and ***: *p* < 0.0001 as compared to blend groups. A significant linear relationship between prediction stability and LogP of API is shown in presence of either (◯) cellulose, silica, and sucrose, (●) mannitol, (●) HPMC or (●) lactose pure excipients. Shapiro–Wilk *p*-value > 0.05. https://www.statskingdom.com/linear-regression-calculator.html (accessed on 25 February 2025).

To further assess the predictive robustness of *Smart Formulation*, Figure 2 illustrates the stability predictions for 15 APIs under different storage conditions and excipient compositions. The model’s performance was evaluated based on the relationship between predicted stability and the LogP of APIs, as well as the influence of excipient selection at varying temperatures and API contents. At 4 °C and 10% API content (Figure 2A), the model identified a significant linear relationship between predicted stability and LogP in the presence of pure excipients such as cellulose, mannitol, silica, and sucrose (*p* < 0.01). Notably, stability predictions for APIs formulated with HPMC and lactose were significantly lower than those with other excipients (*p* < 0.0001), indicating a potential destabilizing effect of these excipients at low temperatures. At 25 °C and 10% API content (Figure 2B), a similar trend was observed, with HPMC and lactose formulations showing significantly lower predicted stability compared to cellulose, silica, sucrose, and mannitol (*p* < 0.01). Interestingly, pure lactose formulations demonstrated a significant positive correlation between LogP and stability, indicating a potential temperature-dependent stabilization effect. Under accelerated conditions at 40 °C (Figure 2C), the influence of excipient selection became more pronounced. Blends containing HPMC, lactose, silica, and sucrose exhibited significantly reduced stabilities (*p* < 0.0001), while pure cellulose, mannitol, silica, and sucrose offered greater stability. This suggests that some excipient blends may exacerbate degradation at high temperatures. Finally, at 25 °C and a higher API content of 90% (Figure 2D), the model again demonstrated a strong correlation between LogP and predicted stability. However, formulations using blended excipients consistently resulted in significantly lower BUDs (*p* < 0.0001), underscoring the importance of excipient selection when scaling up API concentration. This result suggests that more lipophilic APIs tend to exhibit reduced stability in solid formulations, likely due to heterogeneous dispersion within the matrix, unfavorable interactions with residual moisture, and increased sensitivity to oxidation. This relationship is particularly pronounced in formulations containing hygroscopic excipients such as lactose or HPMC. These findings confirm that *Smart Formulation* captures the complex interplay between molecular properties, excipient composition, and storage conditions in predicting API stability. The relationships between LogP, excipient type, and temperature highlight the need for formulation strategies tailored to specific APIs and environmental factors.

**Figure 3:**
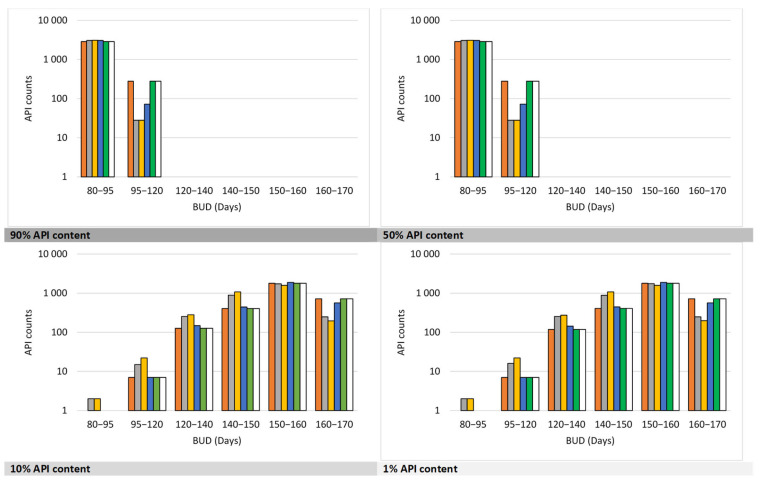
Comparison of BUDs for 3166 APIs (MW: 12.01–1461.43 g.mol^−1^; LogP: −12.01–17.16) formulated in ■ cellulose, ■ HPMC, ■ lactose, ■ mannitol, ■ silica, and ◻ sucrose (API content: 90% to 1%) then stored at 25°C in plastic containers. For API contents of 1%, 10%, 50%, and 90%, significant differences were observed between excipients in the 80–170-day range (*p* < 0.0001). No significant difference was found between excipients in the 80–95-day category (Chi^2^ test).

Additionally, *Smart Formulation* was used to estimate BUDs for a large dataset of 3166 APIs stored at 25 °C in plastic containers across a wide range of molecular weights (12.01–1461.43 g/mol) and LogP values (−12.01–17.16) (Figure 3). The predicted BUDs varied significantly depending on both API content and excipient composition. At high API content (90%), cellulose, mannitol, and silica appeared to promote longer stability, while those with HPMC and lactose resulted in shorter predicted BUDs. Similar trends were observed at 50% API content, although differences between excipients were less pronounced. At 10% and 1% API contents, the model identified significant excipient-related differences in predicted BUDs (80–170 days; *p* < 0.0001). However, within the 80–95 day range, no statistically significant differences were observed (Chi^2^ test), suggesting a possible stabilization threshold beyond which excipient effects diminish.

**Figure 4:**
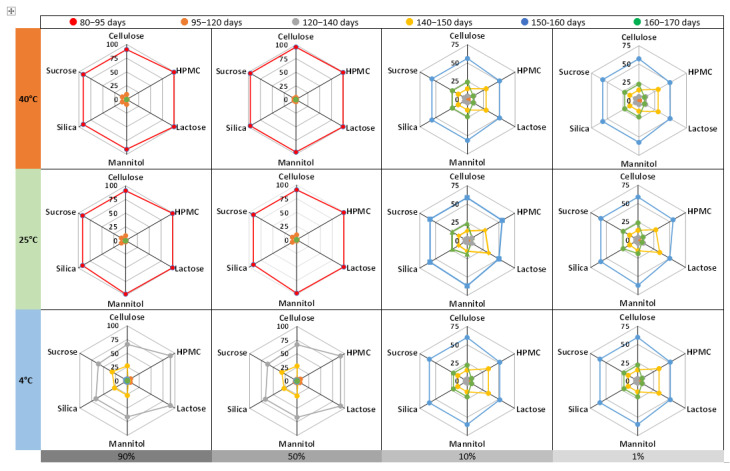
Frequency of formulation categories with BUDs of 80–170 days. Radar charts represent the distribution of formulation categories (% on the *y*-axis) based on their BUD across different excipients (cellulose, HPMC, lactose, mannitol, silica, and sucrose). The formulations are classified according to their API content, ranging from 90% to 1%, and stored at various temperatures (40 °C, 25 °C, and 4 °C). The color-coded categories indicate the frequency of formulations falling within specific BUD ranges. For example, unlicensed preparations formulated with HPMC containing 10% API and stored at 40 °C show the following distribution: 50.5% of formulations have a BUD between 150 and 160 days, 29.5% between 140 and 150 days, 10% between 120 and 140 days, 9% between 160 and 170 days, and 1% between 80 and 120 days. All formulations were packaged in plastic containers.

Figure 4 presents radar charts illustrating the distribution of formulation categories with predicted BUDs between 80 and 170 days, based on API content (90%, 50%, 10%, and 1%), storage temperature (4 °C, 25 °C, and 40 °C), and excipient type. All formulations were packaged in plastic containers. A key observation is that higher API content formulations (90% and 50%) consistently showed shorter BUDs, regardless of storage temperature, suggesting that increased molecular interactions, reduced excipient shielding, and solubility-related degradation may occur at higher concentrations. At 40 °C, most high-content formulations (90% and 50%) fell within the 80–120-day range, while lower API content formulations (10% and 1%) exhibited a broader stability window with many BUDs in the 140–170-day range. These patterns were also seen at 25 °C and 4 °C, reinforcing the importance of dilution effects and excipient interactions in enhancing API stability during storage. Data analysis further indicates that oral solid formulations with a high API content generally exhibit shorter BUDs, regardless of the storage temperature profile (40 °C, 25 °C, or 4 °C). This trend can be attributed to several factors: (i) the reduced proportion of stabilizing excipients, (ii) the increased surface area of the exposed drug, (iii) the lower capacity of the matrix to trap moisture or degradation catalysts, and (iv) changes in the thermodynamic properties of the formulation. Therefore, high drug loading compromises stability by weakening the physicochemical protection mechanisms offered by the excipients. These findings highlight the importance of optimizing API loading in conjunction with appropriate excipient selection to ensure long-term stability.

Table 3 and Table 4 provide a detailed comparison of the stability of 15 and 27 APIs, respectively, across different formulations. These include raw materials, compounded preparations (predicted by *Smart Formulation* with 10% API content at 25 °C, and commercial specialty products (manufacturer data). The stability of these APIs is assessed using various metrics, including the shelf-life of the raw materials, the BUD for compounded preparations, and the expiration date for commercial specialties.

Table 3 compares the stability of different APIs across various formulations. For most APIs, *Smart Formulation* predicts BUDs for compounded preparations that typically range from a few months to about 6 months. *FormulationAI* usually estimates a slightly longer BUD, around 180 days. In contrast, commercial specialty formulations generally have much longer shelf lives, often ranging from 2 to 5 years. This extended stability is primarily due to the substantial number of excipients (EXP) present in these formulations, which help stabilize the API. For example, acetaminophen follows this general trend. *Smart Formulation* estimates a BUD of 156 to 171 days for compounded acetaminophen preparations, while *FormulationAI* predicts a BUD of 180 days. However, for the commercial specialty version of acetaminophen, the shelf-life is significantly longer, reaching up to 5 years. The presence of a higher number of excipients in the specialty formulation likely accounts for this extended stability, which is consistent with the general observations across other APIs in the table. Moreover, the findings also confirm that LogP is a critical determinant of the stability of APIs in solid oral formulations. The increased instability of lipophilic compounds (LogP > 3) in unprotected matrices justifies the widespread use of film coating techniques in industrial products, which are typically absent in hospital compounding, thereby limiting the BUD. This reinforces the value of predictive models incorporating lipophilicity to optimize the shelf-life of compounded preparations.

Building on these findings, Table 4 expands the analysis to include 27 APIs, providing a more comprehensive comparison across various formulations and excipients. This table further emphasizes the role of the excipients, such as lactose and mannitol, in influencing the stability of compounded preparations. It also incorporates formulations in hard capsules, reflecting a broader spectrum of stability across different formulation types. For instance, acetazolamide and cetirizine exhibit varying stability depending on the excipient used, with the predicted BUDs for compounded preparations ranging from 60 to 180 days. The table also compares the BUDs predicted by *Smart Formulation*, those indicated in the MTF, and the expiration dates of raw materials and commercial formulations. For example, the BUDs for compounded acetaminophen preparations are estimated by *Smart Formulation* to be between 156 and 171 days, whereas the MTF indicates a BUD of 180 days. Commercial specialties typically have expiration dates ranging from 2 to 3 years. Interestingly, the BUD of spironolactone (25 mg) compounded in lactose/silica and lactose alone was found to be 60 and 180 days in the MTF and the French National Formulary, respectively, highlighting the impact of mono- and binary excipient combinations on BUD extension. Notably, the BUD of spironolactone (25 mg) in lactose-based preparations, as predicted by *Smart Formulation* (159–164 days; plastic packaging; 25 °C), closely aligned with the French monograph value (https://ansm.sante.fr/uploads/2021/03/25/gelules-de-spironolactone-0-5-a-25-mg.pdf (accessed on 25 February 2025)). Furthermore, *Smart Formulation* could help address gaps in BUD data for unlicensed preparations listed in national formularies. For example, in the French National Formulary, the predicted BUDs for unlicensed preparations of nadolol (1–50 mg) and amiodarone (5–200 mg) in mannitol hard capsules (No. 4 and 2) were 159–168 days and 156–170 days, respectively, at 25 °C in plastic packaging. Moreover, this detailed comparison highlights the differences between raw material shelf-life, BUDs predicted by *Smart Formulation*, and actual expiration dates for commercial specialties, further demonstrating the influence of excipients on stability and the predictive accuracy of *Smart Formulation* when compared to real-world data.

Production conditions for compounded preparations in community and hospital pharmacies are less controlled than in the industrial sector. The crystallization or amorphization of APIs and excipients is typically not monitored, as quality control in solid oral forms primarily focuses on mass and content [40,41]. Similarly, water sorption by APIs and excipients is rarely investigated during compounding activities [42]. However, the revised BUD limit tables in USP-NF <795> have introduced the concept of *water activity* to evaluate the susceptibility of nonsterile preparations to microbial contamination and the potential for degradation through hydrolysis [12]. This lack of physicochemical oversight can lead to variations in BUDs. However, using only one or two excipients simplifies preparation, reducing material controls, procurement delays, and costs—critical advantages amid drug and raw material shortages in Europe. Excipient selection significantly influences stability, solubility, and mechanical properties. Excipients such as cellulose, silica, sucrose, and mannitol provide superior stability, whereas HPMC and lactose, characterized by higher HDB, HAB, and MSC values (Table 2), tend to promote moisture uptake, accelerating API degradation. A clear inverse relationship exists between API hydrophilicity (low LogP) and extended stability, as hydrophilic APIs integrate more effectively into excipient matrices, reducing molecular mobility. Interestingly, formulations with two excipients exhibit systematically shorter BUDs, likely due to disrupted excipient interactions, moisture redistribution, and phase separation effects. Machine learning models integrating experimental stability data and molecular descriptors can enhance excipient-API compatibility predictions, optimizing formulation design and improving compounded preparation stability.

Furthermore, the *Smart Formulation* platform is designed to support decision-making in hospital and pharmacy settings by aligning with existing quality assurance frameworks and regulatory standards (USP-NF <795>/<797>, Ph. Eur.), particularly in situations such as drug shortages where rapid and reliable formulation guidance is crucial.

### 2.3. Web Integration

A key innovation of *Smart Formulation* lies in its integration of excipient variability into stability prediction. Unlike *FormulationAI*, which primarily focuses on API stability, our model allows users to predict stability while considering up to two excipients of their choice. Unlike *FormulationAI*, which centers primarily on active pharmaceutical ingredient (API) stability, *Smart Formulation* enables users to assess stability in the presence of one or two excipients of their choice. This capability is critical, as excipients significantly modulate degradation kinetics, solubility, and overall formulation robustness. By incorporating excipient interactions, the model delivers a more mechanistic and adaptable framework for predicting pharmaceutical stability.

Another differentiating feature is the model’s flexibility regarding environmental conditions. Whereas existing AI-based models often restrict predictions to a limited set of predefined storage scenarios, *Smart Formulation* allows dynamic exploration across a broader spectrum of storage temperatures and packaging configurations. This includes variable container types and closure systems, factors known to influence moisture ingress, oxygen permeability, and light exposure, thereby enhancing the model’s applicability to real-world storage conditions.

In contrast to the categorical outputs typical of current models (e.g., fixed intervals such as “3 months” or “6 months”), Smart Formulation provides time-continuous predictions expressed as beyond use dates (BUDs) with associated standard errors in days. This enables fine-grained decision-making for formulation development, shelf-life assignment, and risk assessment.

The web-based interface (Figure 2B VI Output Data) reflects this user-centric design. Users can input multiple formulation variables, including API identity, excipient composition, storage temperature, packaging material, and API concentration, through a streamlined interface. The output section provides detailed metrics including predicted BUDs, excipient effect magnitude, relevant molecular descriptors, and a confidence-weighted stability classification. This modular architecture makes *Smart Formulation* a practical and scalable tool for pharmaceutical scientists seeking data-driven guidance in formulation optimization.

### 2.4. Limitations and Future Work

One of the main limitations of this study is the relatively small size of the dataset (n = 55), which may affect the generalizability of the model, especially when extrapolated to a broader range of APIs. This limitation is not due to selective inclusion, but rather to the scarcity of publicly available and exploitable stability data for compounded oral solid dosage forms. We have included all experimental BUD data accessible from the literature and databases such as Stabilis, and to our knowledge, no larger curated dataset currently exists in this specific context.

Despite this limitation, the model produced encouraging predictive results and highlighted meaningful trends. In future work, we aim to expand the dataset through collaboration with hospital compounding units and academic partners. Additionally, we are considering synthetic data augmentation techniques to enrich the model and explore predictive performance on a larger scale, while carefully maintaining biological and pharmaceutical relevance.

## 3. Materials and Methods

Data Collection and Advanced Molecular Formulation Database (AMF-DB)

Fifty-five stability data points involving 23 APIs and six excipients, used alone or in combination with one other excipient, were collected from the *Stabilis* database as the primary data source [30]. The dataset covers a range of API content from less than 0.01% to 100% of the total unit dose mass, with storage temperatures spanning from −20 °C to 40 °C. Three types of packaging materials (glass vials, plastic vials, and paper) were included to assess formulation conditioning. The corresponding BUDs were also recorded. The Advanced Molecular Formulation Database (AMF-DB) was built from these experimental data, incorporating (i) common names and canonical simplified molecular input line entry system (SMILES) representations, (ii) formulation-specific information, and (iii) physicochemical molecular descriptors retrieved from public resources, including *ChEMBL* and *PubChem* databases [23,24]. The selected molecular descriptors included molecular weight (MW), n-octanol/water partition coefficient (LogP), number of rotatable bonds (RB), polar surface (PS) hydrogen bond donors (HBD), hydrogen bond acceptors (HBA), and aromatic rings (AR). An overview of the database is provided in Table 5. Similarly, the fundamental properties of excipients were retrieved from the *ChEMBL* and *PubChem* databases [23,24], while their functional roles, as detailed in the Handbook of excipients [43], are summarized in Table 6. Additional information on shelf-life and the crystalline or amorphous nature of excipients was obtained directly from API suppliers.

To optimize machine learning predictions, these molecular descriptors, along with formulation parameters (API content, excipient composition), packaging type, and storage conditions, were systematically categorized to enhance model interpretability and performance. This categorization framework was consistently applied to all molecular and formulation descriptors to ensure consistency and facilitate machine learning model training. MW was divided into 25 classes, increasing in increments of 50 g/mol, ranging from values below 50 g/mol to those exceeding 1200 g/mol. LogP was categorized into 46 classes with 0.5-unit increments, covering values from less than −10 to greater than 12. The number of rotatable bonds (RB) was grouped into 8 classes, increasing by steps of three, from molecules with fewer than three RB to those with more than 20. PS followed a similar approach, classified into 21 classes with increments of 50 Å^2^, spanning values from below 50 Å^2^ to beyond 1000 Å^2^. HBD and HBA were both divided into 8 classes, with 3-unit steps, ranging from values below 3 up to those exceeding 20. AR count was similarly categorized into 8 classes, incrementing by 3 rings, covering molecules with fewer than 3 rings to those with more than 20. API content was classified into 10 levels, increasing by 10% increments, from formulations containing less than 10% API to those exceeding 90%. Packaging material was categorized into three types: glass, plastic, and paper. Temperature stability was divided into 15 classes, increasing in steps of 5 °C, encompassing storage conditions from below −15 °C to above 50 °C. To enhance model interpretability, three composite indices were introduced by aggregating multiple parameters into broader predictive features. The molecule class (MC) was computed as the sum of the MW class, LogP class, and RB class. The molecular structure class (MSC) was obtained by summing up the PS, HBD/HBA, and AR classes. Finally, the storage class was determined by combining the packaging, content, and temperature stability classes.

Excipient composition was encoded using a seven-element binary vector, where the presence or absence of a specific excipient was represented by either a 1 or 0. The excipients lactose, silica, cellulose, mannitol, sucrose, and hydroxypropyl methylcellulose (HPMC) were each assigned specific positions in the vector, with an additional reserved slot left for potential future excipients. For example, a formulation containing only lactose was represented as [1, 0, 0, 0, 0, 0, 0], while a formulation containing both lactose and cellulose was encoded as [1, 0, 1, 0, 0, 0, 0]. This structured encoding system provided a computationally efficient representation of excipient compositions, facilitating their integration into predictive machine learning models for the stability assessment of pharmaceutical formulations.

Several grouping strategies were tested during model development, and the categorization presented here was selected based on its superior performance in terms of classification accuracy and model interpretability. This structure allowed the algorithm to better distinguish between relevant patterns in the dataset.

### 3.1. Smart Formulation Development

**Figure 5:**
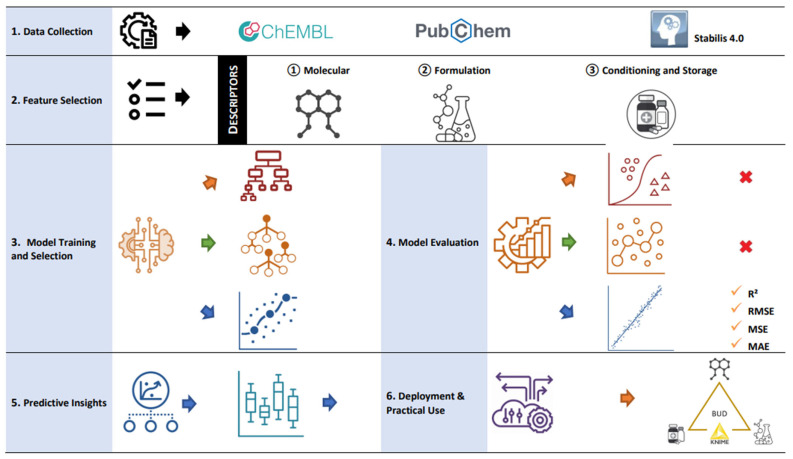
Machine learning workflow used in *Smart Formulation* to predict the beyond use date (BUD) of active pharmaceutical ingredients (APIs) in oral solid dosage forms. The approach relies on a Tree Ensemble Regression Algorithm, a powerful supervised learning method that captures complex non-linear relationships between molecular properties, formulation parameters, and environmental conditions. The model is trained on a dataset comprising three categories of input descriptors: 1. API Descriptors (18 features)—Molecular properties such as molecular weight (MW), logP (lipophilicity), rotatable bonds (RB), polar surface area (PS), hydrogen bond donors (HBD), and acceptors (HBA), among others. 2. Formulation Descriptors (4 features)—Encoded excipient compositions, including lactose, silica, cellulose, mannitol, sucrose, and hydroxypropyl methylcellulose (HPMC), as well as API content percentage. 3. Conditioning and Storage Descriptors (5 features)—Packaging type (glass, plastic, and paper), storage temperature, and classification of storage conditions. The tree ensemble regression algorithm processes these features to establish correlations between molecular properties, formulation parameters, and environmental conditions, ultimately predicting the BUD in days. Notably, the model identifies an inverse correlation between LogP and BUD, suggesting that higher lipophilicity is associated with reduced stability. This predictive approach enables formulators to estimate stability efficiently, reducing reliance on extensive real-time stability studies.

Seven conventional machine learning algorithms were tested to develop the predictive stability model using AMF-DB. These algorithms include the single decision method (decision trees), ensemble methods (random forests, random forest regression, tree ensembles, and tree ensembles regression), and boosting methods (gradient boosted trees and gradient boosted trees regression). By leveraging these diverse methodologies, the model effectively captured complex relationships between formulation parameters, molecular descriptors, and stability outcomes, improving predictive accuracy and robustness across various pharmaceutical formulations. AMF-DB and these algorithms were implemented using the KNIME platform, an open-source data analytics environment widely used in scientific research. KNIME was selected for its ability to support the visual construction of data workflows through a modular interface, which facilitated the development of our machine learning pipeline without requiring extensive coding. This allowed for efficient preprocessing, integration, and the transformation of heterogeneous data sources, including experimental BUD values and molecular descriptors. The platform offers access to multiple machine learning algorithms, enabling systematic comparison and performance optimization using standardized workflows. In our case, it allowed the implementation of a predictive model architecture based on tree ensemble methods. KNIME also facilitated intermediate data analysis, the visualization of descriptor distributions, and performance assessment through embedded tools. The entire workflow used in this study is documented to ensure reproducibility and transparency, and the main steps of the process are illustrated in Figure 5. While several other platforms could have been used, KNIME was chosen for its compatibility with the project requirements and for ensuring consistency in data handling across all stages of model development.

The detailed configuration of the machine learning workflow, including the list of nodes and hyperparameter settings used for the tree ensemble regression model, is provided in Supplementary File S1.

### 3.2. Model Evaluation

**Figure 6:**
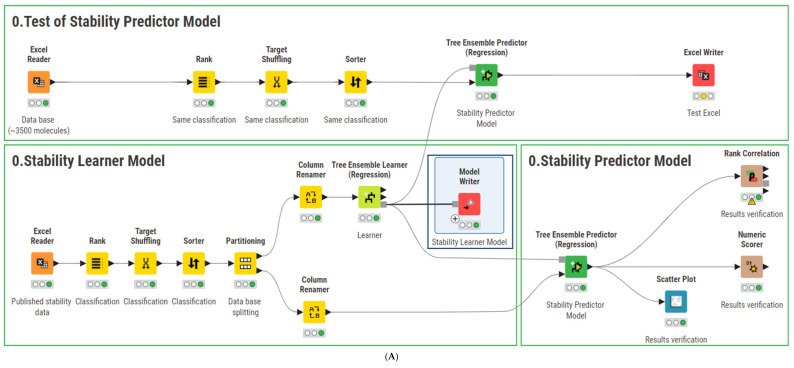

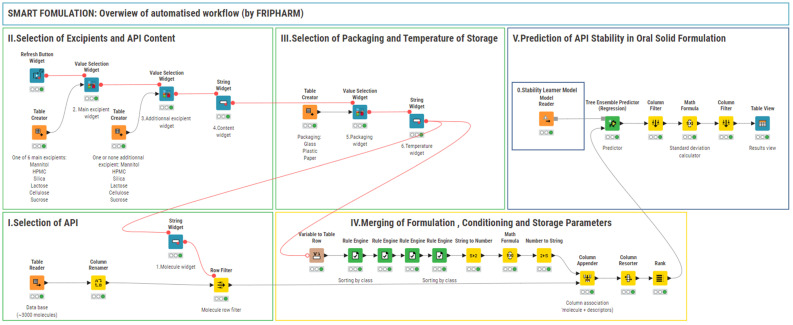

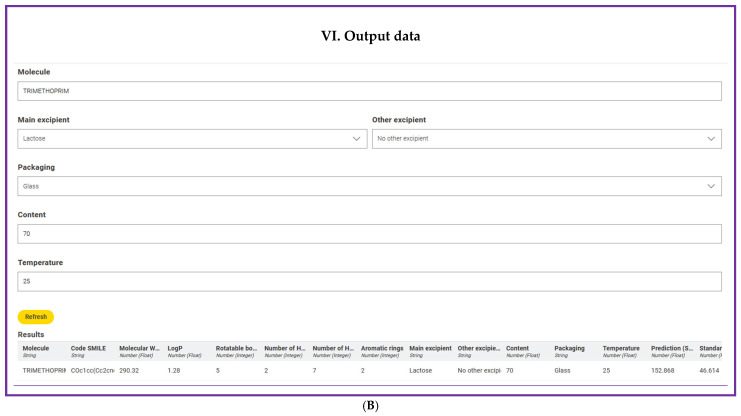
(**A**) Predictive stability model of API using Tree Ensemble Regression learner algorithm in KNIME. (**B**) Automated workflow deployment for API stability prediction in compounded oral solid preparations, considering excipients, API content, packaging, and storage temperature. *VI Output* data *Panel* must be filled in https://www.knime.com/smart-formulation-data-app (accessed on 11 April 2025).

Figure 6A illustrates a representative workflow of *Smart Formulation* developed using AMF-DB and the tree ensemble regression algorithm. In the “Partitioning” node, the dataset was divided into subsets using a linear selection approach, with 80% allocated to the training set. The learner node uses the “BUD” column (Table 5) as the target variable to train the model, which is then applied to the remaining 20% of the data in the predictor node. The “Scorer” node evaluates the results using a confusion matrix [28]. These subsets were integrated into the seven machine learning learner nodes. To ensure the reliability of predictions, the following statistical metrics were employed: coefficient of determination (R^2^), mean absolute error (MAE), mean squared error (MSE), root mean squared error (RMSE), mean signed difference (MSD), and mean absolute percentage error (MAPE). All evaluation metrics were computed using the “Numeric Scorer” node in KNIME, enabling a systematic and standardized assessment of model performance. Additionally, statistical analyses were conducted to assess correlations between molecular descriptors, formulation composition, conditioning parameters, storage conditions, and the predicted BUD of compounded APIs.

### 3.3. Model Validation and Performance Testing

To assess the generalizability of Smart Formulation, three independent validation tests were conducted using APIs not included in the training dataset. The first test set consisted of 15 APIs (MW: 129.17 to 776.87 g.mol^−1^, LogP: −0.92 to 5.39; initial content: 10%; packaging material: plastic), formulated in six pure excipients and 15 different binary excipient combinations. Predicted BUDs were compared across three reference conditions: (i) APIs as raw materials (supplier data), (ii) APIs in compounded preparations (predicted by *Smart Formulation* at 10% content, 25 °C, and by *FormulationAI*), and (iii) APIs in commercial specialties (manufacturer data). A second independent test set included 27 APIs, where stability predictions were evaluated in three settings: (i) APIs as raw materials (supplier data), (ii) APIs in unlicensed preparations (predicted by *Smart Formulation* at 10% content, 25 °C, and reported in the Magistral Therapeutic Formulary [32]), and (iii) APIs in licensed products (manufacturer data). Finally, 3160 APIs (MW: 12.01 to 1461.43 g.mol^−1^; LogP: −12.01 to 17.16) were analyzed to assess the influence of excipients and storage conditions on stability. These APIs were formulated in **six pure excipients** (API content: 90% to 1%) and stored at 40 °C, 25 °C, and 4 °C. The physicochemical properties and therapeutic classification of 3166 APIs are detailed in Table 7. This multi-layered validation strategy provided a comprehensive assessment of the model’s predictive accuracy across diverse pharmaceutical formulations and storage conditions.

### 3.4. Web Integration

In this study, a KNIME workflow was used to integrate molecular descriptors, formulation parameters, and storage conditions to assess the BUDs of 3166 API-based unlicensed preparations. These APIs were formulated either with one of six pure excipients or with binary mixtures of two excipients and packaged in three types of containers. Considering four API content levels (1%, 10%, 50%, and 90%) and three storage temperatures (4 °C, 15 °C, and 40 °C), the total number of potential BUD predictions is estimated at approximately 1,700,000. Figure 6B presents a representative automated workflow of *Smart Formulation*, offering an interactive visualization of the data output.

## 4. Conclusions

This study demonstrates the feasibility of applying machine learning techniques to predict the stability of APIs in oral solid dosage forms. The tree ensemble regression algorithm effectively correlates molecular descriptors and formulation parameters with BUD, underscoring the pivotal influence of lipophilicity, particularly LogP, on degradation kinetics. The resulting model offers a predictive tool of high practical value for pharmaceutical development, presenting a cost-effective and time-efficient alternative to conventional long-term stability testing. These findings are consistent with recent advances in computational pharmaceutics that seek to bridge theoretical formulation strategies with real-world applications. By embedding artificial intelligence into the formulation design process, *Smart Formulation* provides a robust framework to enhance prediction accuracy, streamline resource allocation, and strengthen the stability of extemporaneous preparations. Its integration into both community and hospital pharmacy workflows holds the potential to mitigate drug shortages, standardize compounding practices, and improve overall patient safety. Looking ahead, future developments may include real-time stability monitoring systems and adaptive machine learning models capable of refining predictions based on continuous data acquisition. These advancements would further consolidate Smart Formulation’s role as a cornerstone innovation in the evolving field of personalized, AI-assisted pharmaceutical formulation.

The *Smart Formulation* platform is already available online and can be accessed by professionals upon request. While it is not publicly indexed, access is granted via direct link to support its use as a decision-support tool in hospital, academic, and compounding pharmacy settings.

## Figures and Tables

**Table 2:** Correlation between molecular descriptors, formulation, conditioning, storage parameters, and predicted BUD of compounded APIs as obtained with Tree Ensembles Regression model. Significant correlations (*p* < 0.05) are highlighted in gray.

Parameters	Correlation Value	*p*-Value
Molecular descriptors		
Molecule	−0.269	0.204
Code SMILE	−0.163	0.447
MW	0.305	0.148
MW class	0.089	0.678
LogP	0.503	0.012
LogP class	0.502	0.012
Rotatable bonds	0.087	0.686
Rotatable bonds class	0.157	0.464
Polar surface	0.319	0.129
Polar surface class	−0.153	0.476
H-donor bonds	−0.183	0.392
H donor bonds class	−0.271	0.201
H-acceptor bonds	−0.162	0.449
H acceptor bonds class	−0.128	0.550
Aromatic rings	0.357	0.087
Aromatic rings class	0.283	0.180
Molecule class	0.439	0.032
Molecular structure class	−0.139	0.517
Formulation descriptors		
Main excipient	0.041	0.849
Encoded excipients	0.300	0.154
Content	−0.212	0.321
Content class	−0.269	0.203
Conditioning and storage descriptors		
Packaging	−0.200	0.349
Packaging class	0.145	0.500
Temperature	−0.336	0.109
Temperature class	−0.110	0.608
Storage class	−0.266	0.209

**Table 3:** Comparative stability assessment of fifteen active pharmaceutical ingredients across three conditions: as raw materials (based on supplier data), in compounded oral preparations (predicted by *Smart Formulation* under standard conditions: 10% content, 25 °C), and in marketed pharmaceutical products (manufacturer-reported data). For each active ingredient, the predicted BUD is presented alongside relevant formulation variables. Sources of raw material data include *^a^*: Inresa; *^b^*: Cerata Pharmaceuticals LL; *^c^*: Newstar Chem Enterprise LTD; *^d^*: Neutralpharma; *^e^*: Nicolas Green Pharmaceuticals; *^f^*: Xi’An Tian Guangyuan Biotech Co., Ltd.; ^‡^: Vidal Hoptimal, and ^¥^: HPMC. *Smart Formulation* predictions are compared to outputs *from FormulationAI*, which estimates physical stability in mannitol and HPMC matrices under identical storage conditions. The physical state of the active ingredient is indicated as crystalline (C) or amorphous (A) based on the predominant solid form. EXP: excipient.

API	LogP	Shelf-Life (Raw Material)	BUD (Preparation)			Expiration Date (Specialty) ^‡^	Film Coated Tablet
			1 EXP	2 EXP	*FormulationAI*		
Acetaminophen (C)	0.91	4 years; 15–25 °C *^a^*	156–170 days	156–171 days	180 days	5 EXP/5 years *Claradol 500 mg*	No
Amlodipine (besylate) (C)	1.64	5 years; 15–25 °C *^a^*	157–165 days	148–153 days	180 days	3 EXP/3 years *Amlodipine 10 mg*	No
Aspirin(C)	1.24	3 years; 15–25 °C *^a^*	166–174 days	163–173 days	180 days	2 EXP/3 years *Aspirine du Rhône 500 mg*	No
Atorvastatin (calcium) (A)	5.39	5 years; 15–25 °C *^b^*	136–149 days	134–141 days	180 days	6 EXP/2 years *Atorvastatin 80 mg*	Yes
Clarithromycin (C)	3.24	3 years; 15–25° C *^c^*	164–170 days	155–158 days	180 days	7 EXP/3 years *Clarithromycine 250 mg*	Yes
Diazepam(C)	3.08	5 years; 15–25 °C *^a^*	155–168 days	149–154 days	180 days	4 EXP/3 years *Diazepam 10 mg*	No
Fluoxetine (Chlorhydrate) (C)	1.7	5 years; 15–25 °C *^d^*	144–158 days	141–147 days	180 days	3 EXP/3 years *Fluoxetine 20 mg*	Dispersible
Hydrochlorothiazide (C)	−0.58	5 years; 15–25 °C *^a^*	161–174 days	161–174 days	180 days	5 EXP/3 years *Esidrex 25 mg*	No
Ibuprofen(C)	3.84	5 years; 15–25 °C *^a^*	156–170 days	150 days	180 days	11 EXP/3 years *Advil 200 mg*	Yes
Levothyroxine (sodium) (A)	−2.3	2 years; 15–25 °C *^f^*	155–171 days	150–160 days	180 days	5 EXP/3 years *Thyrofix 100 µg*	No
Losartan (potassium) (C)	4.06	5 years; 15–25 °C *^b^*	154–165 days	151–156 days	180 days	7 EXP/3 years *Losartan 50 mg*	Yes
Metformin (hydrochloride) (C)	−0.92	5 years; 15–25 °C *^e^*	164–177 days	164–177 days	180 days	4 EXP/5 years *Glucophage 500 mg*	No
Omeprazole (C)	2.43	2 years; 15–25 °C *^a^*	157–171 days	150–160 days	180 days	8 EXP/3 years *Omeprazole 10 mg*	Enteric film
Prednisolone (C)	1.27	3 years; 15–25 °C *^a^*	156–171 days	148–157 days	180 days	4 EXP/3 years *Prednisolone 20 mg*	No
Simvastatin (A)		3 years; 15–25 °C *^a^*	151–163 days	149–155 days	180 days /Instable ^¥^	11 EXP/2 years *Simvastatine 40 mg*	Yes

**Table 4:** Stability comparison of 27 APIs across three conditions: as raw materials (based on supplier data), in compounded preparations (predicted using the *Smart Formulation* model under standard conditions: 10% at 25 °C, or as reported in the Magistral Therapeutic Formulary), and in commercial pharmaceutical specialties (according to manufacturer data). For each active ingredient, the predicted or reported BUD is presented alongside relevant formulation parameters. Sources of raw material data include *^a^*: Inresa; *^b^*: Metrix Food And Pharma *^c^*: Pharmacompass (www.pharmacompass.com (accessed on 25 February 2025)); *^d^*: Samex Overseas; *^e^*: Yihuipharm; *^f^*: Saliuspharma ^‡^: Vidal Hoptimal. Magistral Therapeutic Formulary (MTF). Diluent A: Lactose/silica; diluent B: Mannitol/silica. Hard capsule volume: #000: 1.36 mL; #00: 0.95 mL; #0: 0.67 mL; #1: 0.48 mL; #2: 0.37 mL; #3: 0.27 mL. NA: non-available. EXP: excipient.

API (API Content %)	Shelf-Life (Raw Material)	BUD (Preparation)			Expiration Date (Specialty) ^‡^
		1 EXP	2 EXP	MTF	
Acetazolamide 250 mg (>50%)	3–4 years; 15–25 °C *^b^*	Lactose G: 165 Pl: 167 P: 162 days	Lactose/silica G: 173 Pl: 175 P: 170 days	60 days Lactose/silica Hard capsules n° 2	4 EXP/3 years *Diamox 250 mg*
Cetirizine dichlorhydrate 10 mg (<50%)	5 years; 15–25 °C *^a^*	Lactose G: 148 Pl: 148 P: 145 days	Lactose/silica G: 162 Pl: 163 P: 159 days	60 days Lactose/silica Hard capsules n° 3	5 EXP/4 years *Cetirizine 10 mg*
Chenodesoxycholic acid 250 mg (<50%) (>50%)	NA	Mannitol G: 170 Pl: 171 P: 166 days Mannitol G: 160 Pl: 161 P: 157 days	Mannitol/silica G: 154 Pl: 155 P: 150 days Mannitol/silica G: 146 Pl: 147 P: 143 days	180 days Mannitol/silica	NA
Cholecalciferol 100.000 U.I/g 4, 0 mg (<50%)	NA	Lactose G: 109 Pl: 110 P: 110 days	-	60 days Lactose	NA
Clindamycine 150, 300 mg Clindamycine phosphate 163.5, 327 mg (<50%)	2 years; 15–25 °C *^c^*	Lactose G: 164 Pl: 166 P: 162 days Mannitol G: 171 Pl: 174 P: 169 days	Lactose/silica G: 170 Pl: 172 P: 168 days Mannitol/silica G: 163 Pl: 166 P: 161 days	60 days Lactose/silica Hard capsules n° 0 60 days Mannitol/silica Hard capsules n° 0	4 EXP/3 years *Clindamycine 150, 300 mg*
Diosmine 500 mg (<50%)	NA	Lactose G: 142 Pl: 145 P: 144 days	Lactose/silica G: 154 Pl: 157 P: 155 days	60 days Lactose/silica Hard capsules n° 000	7 EXP/3 years *Diosmine 600 mg*
Domperidone 10 mg (<50%)	NA	Lactose G: 162 Pl: 164 P: 159 days	Lactose/silica G: 172 Pl: 174 P: 169 days	60 days Lactose/silica Hard capsules n° 3	10 EXP/3 years *Domperidone Arrow 10 mg*
Doxycycline 50, 100 mg Doxycycline hyclate 58, 116 mg (<50%)	3 years; 15–25 °C *^c^*	Lactose G: 167 Pl: 170 P: 165 days Mannitol G: 175 Pl: 178 P: 173 days	Lactose/silica G: 175 Pl: 178 P: 173 days Mannitol/silica G: 167 Pl: 170 P: 165 days	60 days Lactose/silica Hard capsules n° 1 60 days Mannitol/silica Hard capsules n° 1	10 EXP/3 years *Doxy 50 mg, 100 mg*
Folic acid 0.4, 4 mg (<50%)	NA	Mannitol G: 173 Pl: 176 P: 171 days	Mannitol/silica G: 165 Pl: 168 P: 163 days	60 days Mannitol/silica	5 EXP/30 months 0.4 mg 5 EXP/2 years 5 mg
Fludrocortisone acetate 0.025, 0.050, 0.1 mg (<50%)	NA	Mannitol G: 175 Pl: 177 P: 173 days	Mannitol/silica G: 170 Pl: 173 P: 168 days	60 days Mannitol/silica Hard capsules n° 2	3 EXP/3 years *Flucortac* 50 µg 6 EXP/2 years *Flucortac 0.1 mg*
Furosemide 1 mg à 10 mg (<50%)	NA	Lactose G: 152 Pl: 154 P: 149 days	Lactose/silica G: 165 Pl: 167 P: 161 days	60 days Lactose/silica	4 EXP/3 years *Furosemide 20 mg*
Hydrocortisone 10, 20 mg (<50%)	2–5 years; 15–25 °C *^d^*	Mannitol G: 140 Pl: 141 P: 136 days	Mannitol/silica G: 126 Pl: 127 P: 122 days	60 days Mannitol/silica Hard capsules n° 2	4 EXP/3 years *Hydrocortisone 10 mg*
Loperamide chlorhydrate 2 mg (<50%)	2 years; 2–8 °C *^e^*	Lactose G: 147 Pl: 148 P: 144 days	Lactose/silica G: 160 Pl: 160 P: 156 days	60 days Lactose/silica Hard capsules n° 3	3 EXP/3 years *Diaretyl 2 mg*
Mebeverine chlorhydrate 135 mg (<50%)	NA	Lactose G: 159 Pl: 160 P: 157 days	Lactose/silica G: 167 Pl: 169 P: 167 days	60 days Lactose/silica Hard capsules n° 0	4 EXP/3 years *Mebeverine 100 mg*
Menadione sodium bisulfite 1 mg (<50%)	NA	Mannitol G: 105 Pl: 106 P: 104 days	Mannitol/silica G: 89 Pl: 90 P: 87 days	60 days Mannitol/silica	NA
Minocycline chlorhydrate dihydrate 58, 116 mg (<50%)	NA	Mannitol G: 173 Pl: 174 P: 169 days	Mannitol/silica G: 165 Pl: 166 P: 161 days	60 days Mannitol/silica Hard capsules n° 1	1 excipient/2 years *Minocyne 100 mg*
Primaquine phosphate 30 mg (<50%)	NA	Mannitol G: 160 Pl: 162 P: 159 days	Mannitol/silica G: 147 Pl: 148 P: 145 days	180 days Mannitol/silica	10 EXP/3 years *Primaquine 15 mg*
Pyridoxal phosphate 10 mg (<50%)	5 years; 15–25 °C *^a^*	Mannitol G: 161 Pl: 164 P: 161 days	Mannitol/silica G: 149 Pl: 152 P: 149 days	180 days Mannitol/silica	NA
Ranitidine 150 mg Ranitidine chlorhydrate 167.5 mg (<50%)	36 months; 15–25 °C *^f^*	Lactose G: 155 Pl: 158 P: 153 days	Lactose/silica G: 167 Pl: 169 P: 164 days	60 days Lactose/silica Hard capsules n° 00	8 EXP/3 years *Ranitine EG 150 mg*
Retinol acetate 325.000 U.I/g 12.3 mg (<50%)	2 years; 15–25 °C [39]	Lactose G: 154 Pl: 156 P: 150 days	-	60 days Lactose	NA
Riboflavine 400 mg (<50%) (>50%)	4 years; 15–25 °C *^a^*	Lactose G: 165 Pl: 166 P: 161 days Lactose G: 153 Pl: 155 P: 150 days	Lactose/silica G: 174 Pl: 175 P: 170 days Lactose/silica G: 164 Pl: 165 P: 160 days	60 days Lactose/silica	NA
Scopolamine butylbromure 10 mg (<50%)	NA	Lactose G: 149 Pl: 152 P: 149 days	Lactose/silica G: 164 Pl: 166 P: 163 days	60 days Lactose/silica	NA
Simvastatin 5, 20, 40 mg (<50%)	3 years; 15–25 °C *^a^*	Lactose G: 157 Pl: 157 P: 153 days	Lactose/silica G: 167 Pl: 167 P:164 days	60 days Lactose/silica Hard capsules n° 2	11 EXP/2 years *Simvastatine* *Accord 10, 20, 40 mg*
Spironolactone 25 mg (<50%)	5 years; 15–25 °C *^a^*	Lactose G: 162 Pl: 164 P: 159 days	Lactose/silica G: 172 Pl: 174 P: 169 days	60 days Lactose/silica	5 EXP/18 months *Aldactone 25 mg*
Sulpiride 50 mg (<50%)	NA	Lactose G: 138 Pl: 138 P: 138 days	Lactose/silica G: 149 Pl: 150 P:150 days	60 days Lactose/silica	4 EXP/2 years *Dogmatil 50 mg*
Triamcinolone 4 mg (<50%)	NA	Mannitol G: 158 Pl: 158 P: 156 days	Mannitol/silica G: 149 Pl: 149 P: 147 days	60 days Mannitol/silica Hard capsules n° 2	NA
Trimethoprime 50 mg (<50%)	NA	Lactose G: 163 Pl: 165 P: 160 days Mannitol G: 172 Pl: 174 P: 169 days	Lactose/silica G: 172 Pl: 174 P: 169 days Mannitol/silica G: 163 Pl: 165 P: 160 days	60 days Lactose/silica Hard capsules n° 3 60 days Mannitol/silica Hard capsules n° 3	5 EXP/3 years *Delprim 300 mg*
Trimethoprime 300 mg (>50%)		Lactose G: 153 Pl: 155 P: 151 days Mannitol G: 163 Pl: 166 P: 161 days	Lactose/silica G: 163 Pl: 166 P: 161 days Mannitol/silica G: 153 Pl: 155 P: 151 days	60 days Lactose/silica Hard capsules n° 1 60 days Mannitol/silica Hard capsules n° 1	

**Table 5:** Dataset of (i) molecular descriptors (*ChEMBL*, *PubChem*, and *Drugbank*) of 22 APIs, and (ii) 53 stability data of compounded oral preparation extracted from *Stabilis* and FRIPHARM data. BUD: Beyond use date. Cond.: conditioning; G: Glass; Pl.: plastic; P: paper. ND: not documented. T: temperature; MW: molecular weight (g/mol), RB: rotatable bonds, PS: polar surface (Å^2^), HBD: hydrogen bond donor count, HBA: hydrogen bond acceptor count, AR: aromatic rings, MC: molecule class, MSC: molecular structure class.

API	MW	LogP	RB	PS	HBD	HBA	AR	MC	MSC	Excipients		Cond.	Content		T	BUD
										Main	Other		(mg)	(%)	°C	(Days)
Acetylsalicylic acid	180.16	1.24	2	63.60	1	3	1	30	5	Lactose	-	ND	4	4	25	365
													19	4	25	365
													56	4	25	365
													76	4	25	365
Alpha-tocopherol acetate	472.74	10.42	13	35.53	0	3	1	53	9	Lactose	-	G	100	56	8	60
													100	56	25	60
4-Aminopyridine	94.12	−0.07	1	38.91	1	2	1	24	4	Lactose	Silica	Pl	5	2	25	180
													5	2	40	30
3,4-Diaminopyridine	109.13	−0.9	0	64.93	1	2	1	25	5	Lactose	Silica	Pl	5	2	4	180
													5	2	25	180
Amiodarone Hydrochloride	681.78	7.64	11	42.68	1	4	3	52	9	Cellulose	-	ND	5	2	25	30
													20	10	25	30
													50	25	25	30
										Mannitol	-	G	10	4	25	365
													60	25	25	365
													100	50	25	365
Amoxicillin trihydrate	365.41	−2.31	4	132.96	4	6	1	28	8	-	-	Pl	125	100	25	90
													250	100	25	56
													250	100	40	56
													500	100	25	90
Atenolol	266.34	0.43	6	84.58	2	5	1	30	7	Cellulose	-	PI	25	50	30	120
Captopril	217.29	0.28	3	95.00	2	4	0	29	6	Lactose	-	P	2	2	25	84
Carbidopa	244.24	−1.21	4	115.81	5	5	1	27	7	Cellulose	-	ND	200	30	25	336
Cholecalciferol	384.64	7.13	6	20.53	1	1	0	45	6	Lactose	-	G	0.025	0.008	8	60
													0.025	0.008	25	60
Cholic acid	408.57	2.48	4	97.99	3	5	0	37	7	Silica	Lactose	Pl	25	95	25	365
										Silica	-	Pl	250	97	25	365
										Silica	Lactose	Pl	25	100	40	180
										Silica	-	PI	250	100	40	180
Clonidine hydrochloride	266.56	2.49	1	36.42	2	3	1	33	5	Cellulose	-	ND	0.02	1	25	365
Cyclo- phosphamide	261.09	0.10	5	41.57	1	2	0	29	5	Lactose	-	ND	10	-	4	70
													25	-	4	70
Erythromycin	733.94	2.60	7	193.91	5	14	0	46	11	Cellulose	-	ND	20	46	25	365
Fludrocortisone acetate	422.49	1.76	3	110.90	2	6	0	37	7	Lactose	-	ND	0.01	-	25	180
										Cellulose	-	ND	0.01	-	25	180
										Sucrose	-	ND	0.01	-	25	180
Hydrocortisone	362.47	1.61	0	94.00	3	5	1	35	6	Lactose	-	P	20	0.4	25	12
Melatonin	232.28	1.15	4	54.12	2	4	2	31	6	Lactose	HPMC	G	3	0.7	25	90
										Lactose	HPMC	G	3	0.7	40	90
										Cellulose	-	ND	0.5	0.65	25	547
										Cellulose	-	ND	2	2.6	25	547
										Cellulose	-	ND	6	8.4	25	547
										Lactose	-	P	18	0.7	25	90
										Lactose	-	P	18	0.7	40	90
										Lactose	-	P	18	2	-20	168
Menadione	172.18	1.89	0	34.14	0	2	1	30	4	Lactose	-	G	1	0.5	8	60
										Lactose	-	G	1	0.5	25	60
Midazolam Hydrochloride	362.20	3.97	1	30.18	0	3	3	38	6	Cellulose	-	ND	1	1	25	365
Naltrexone	341.42	1.36	2	70.00	2	5	1	33	5	Cellulose	-	G	1.5	10	25	360
Nifedipine	346.34	2.56	4	107.00	1	6	1	37	7	Lactose	-	P	1	0.2	6	365
										Lactose	-	P	1	0.2	22	365
Retinyl acetate	328.50	5.14	6	26.30	0	2	0	40	6	Lactose	-	G	5.5	1	8	60
										Lactose	-	G	5.5	1	25	60

**Table 6:** Molecular descriptors (*ChEMBL* and *PubChem*), functional categories and properties (adapted from [36]) of excipients. MW: molecular weight (g/mol), RB: rotatable bonds, PS: polar surface (Å^2^), HBD: hydrogen bond donor count, HBA: hydrogen bond acceptor count, AR: aromatic rings, MC: molecule class, MSC: molecular structure class, (C): crystalline as major form; (A): amorphous as major form.

Excipients	Physicochemical Properties									Functional Category		Shelf-Life ^†^ (15–25 °C)
	MW	LogP	RB	PS	HBD	HBA	AR	MC	MSC	Key Functional Roles	Notable Properties	
Cellulose (C) ^a^	342.30	−5.40	4	190.00	8	11	0	22	10	Adsorbent, disintegrant, binder, diluent	Hygroscopic, used in wet/dry granulation	4 years
HPMC ^b^ (A)	1261.40	−2.32	30	365.00	8	30	0	50	20	Dispersing, solubilizing, stabilizing, thickening, film-coating, binder	Nonionic, used in extended-release tablets and film coatings	3 years
Lactose (C/A)	360.31	−5.73	4	191.00	9	12	0	22	12	Binder, filler, diluent	Exists in different crystalline forms	3 years
Mannitol (C)	182.17	−3.73	2	131.38	6	6	0	21	9	Diluent, plasticizer	Non-hygroscopic; suitable for moisture-sensitive APIs.	5 years
Silica ^c^ (A)	60.84	−0.62	0	34.10	2	0	0	23	4	Adsorbent, disintegrant, thermal stabilizer	Hygroscopic; widely used in oral formulations	5 years
Sucrose (C)	342.3	−4.53	5	189.55	8	11	0	40	10	Binder, filler	Stable at room temperature; absorbs ~1% moisture.	5 years

^†^: Inresa sources, ^a^: Cellulose microcrystalline Ph 102, ^b^: Hypromellose 4000, ^c^: Anhydrous colloidal silica.

**Table 7:** Physicochemical properties and therapeutic classification of 3166 APIs. Adapted from the *ChEMBL* database.

API	%
MW (g.mol^−1^)	
<250	20
250–500	62
>500	18
Total	100
LogP	
<0	22
0–2	26
2–4	32
>4	20
Total	100
ATC	
Nervous system	17
Alimentary tract and metabolism	12
Cardiovascular system	12
Anti-infectives for systemic use	11
Dermatologicals	8
Antineoplastic, immunomodulating agents	8
Respiratory system	8
Various	6
Sensory organs	6
Genito urinary system	5
Other	7
Total	100

## Data Availability

The datasets were derived from sources in the public domain, including the following: ***Stabilis* Database** (https://www.stabilis.org (accessed on 25 February 2025)): A reference platform for drug stability, used for extracting experimental BUD data. ***PubChem*** (https://pubchem.ncbi.nlm.nih.gov (accessed on 25 February 2025)): For molecular structure and descriptor data of active pharmaceutical ingredients. ***DrugBank*** (https://go.drugbank.com (accessed on 25 February 2025)): For physicochemical and pharmacological data on APIs. ***PubMed*** (https://pubmed.ncbi.nlm.nih.gov (accessed on 25 February 2025)): For literature-derived stability data and formulation context. ***ChemBL*** (https://www.ebi.ac.uk/chembl/ (accessed on 25 February 2025)): For bioactivity and structural information related to APIs. ***Vidal Hoptimal^®^*** (https://www.vidal.fr/hoptimal (accessed on 25 February 2025)): For pharmaceutical formulation components and hospital-use references. All data were used in accordance with the terms of use of the respective databases and are publicly accessible at the time of this publication. Where applicable, data integration and curation were performed to standardize molecular descriptors and formulation parameters for model training and validation.

## Supplementary Materials

The following supporting information can be downloaded at: https://www.mdpi.com/article/10.3390/ph18081240/s1. File S1: Overview of the learner node settings and hyperparameters applied in the KNIME workflow.
