# Supplementary material for: Smart Formulation: AI-Driven Web Platform for Optimization and Stability Prediction of Compounded Pharmaceuticals Using KNIME
Source: Pharmaceuticals (Basel). 2025 Aug 21;18(8):1240. doi: 10.3390/ph18081240 (PMC12389346; doi:10.3390/ph18081240)
Supplement: Supplementary file 1 [file pharmaceuticals-18-01240-s001.zip › pharmaceuticals-3740758-supplementary.pdf]

Overview of the learner node settings and hyperparameters applied in the KNIME workflow

Nodes	Correspondences
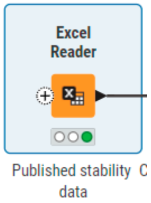	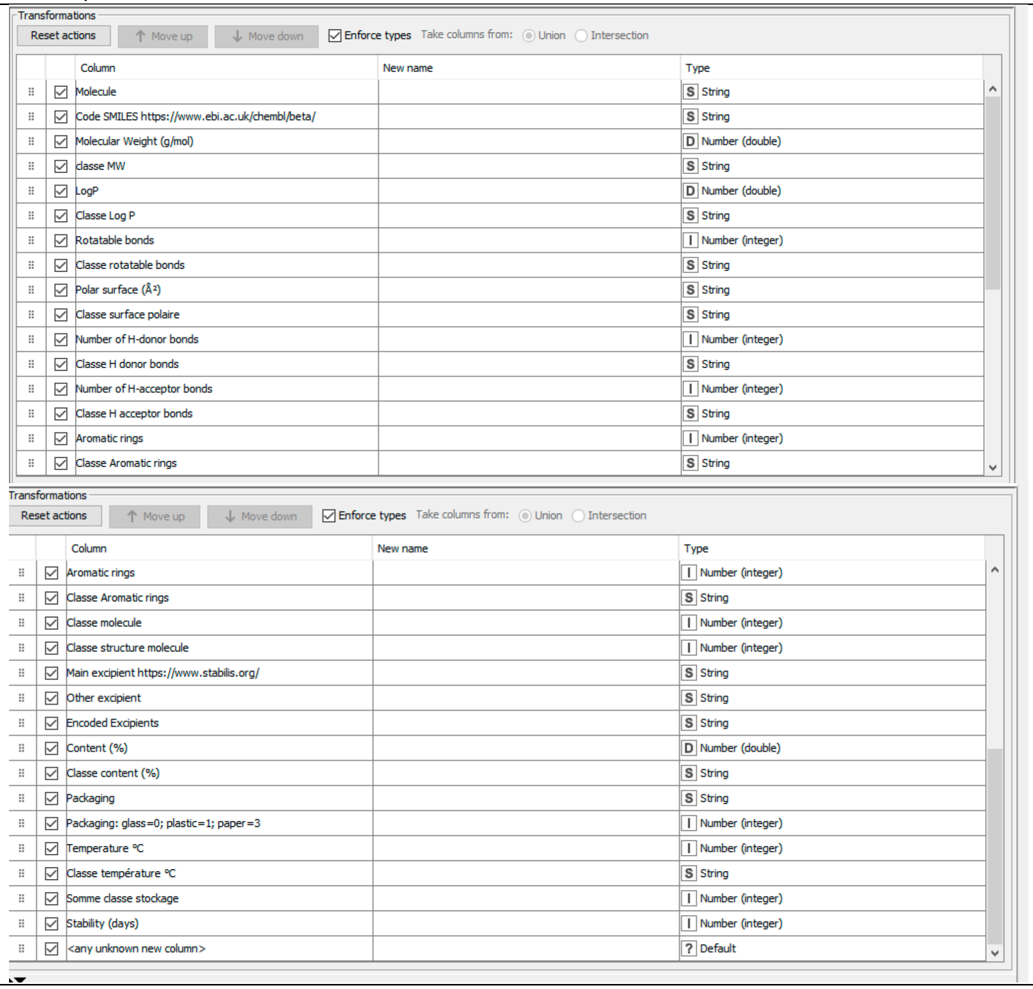

Transformations

Reset actions | Move up | Move down | Enforce types | Take columns from: Union | Intersection

	Column	New name	Type
☑	Molecule		S String
☑	Code SMILES https://www.ebi.ac.uk/chembl/beta/		S String
☑	Molecular Weight (g/mol)		D Number (double)
☑	classe MW		S String
☑	LogP		D Number (double)
☑	Classe Log P		S String
☑	Rotatable bonds		I Number (integer)
☑	Classe rotatable bonds		S String
☑	Polar surface ($Å^2$)		S String
☑	Classe surface polaire		S String
☑	Number of H-donor bonds		I Number (integer)
☑	Classe H donor bonds		S String
☑	Number of H-acceptor bonds		I Number (integer)
☑	Classe H acceptor bonds		S String
☑	Aromatic rings		I Number (integer)
☑	Classe Aromatic rings		S String

Transformations

Reset actions | Move up | Move down | Enforce types | Take columns from: Union | Intersection

	Column	New name	Type
☑	Aromatic rings		I Number (integer)
☑	Classe Aromatic rings		S String
☑	Classe molecule		I Number (integer)
☑	Classe structure molecule		I Number (integer)
☑	Main excipient https://www.stabilis.org/		S String
☑	Other excipient		S String
☑	Encoded Excipients		S String
☑	Content (%)		D Number (double)
☑	Classe content (%)		S String
☑	Packaging		S String
☑	Packaging: glass=0; plastic=1; paper=3		I Number (integer)
☑	Temperature °C		I Number (integer)
☑	Classe température °C		S String
☑	Somme classe stockage		I Number (integer)
☑	Stability (days)		I Number (integer)
☑	<any unknown new column>		? Default

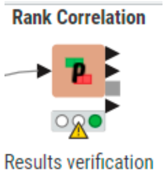

First column name (String)	Second column name (String)	Correlation value (Number (double))	p value ↓ (Number (double))
First column name	2 selected	Correlation value	p value
Temperature	Prediction (Stability (days))	-0.336	0.109
Encoded Excipients	Stability (days)	0.352	0.091
Aromatic rings	Prediction (Stability (days))	0.357	0.087
Classe molecule	Prediction (Stability (days))	0.439	0.032
Classe molecule	Stability (days)	0.476	0.019
Classe Log P	Stability (days)	0.487	0.016
LogP	Stability (days)	0.491	0.015
Classe Log P	Prediction (Stability (days))	0.502	0.012
LogP	Prediction (Stability (days))	0.503	0.012
Stability (days)	Prediction (Stability (days))	0.896	0

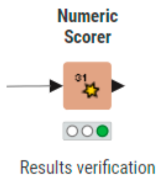

RowID	Prediction (Stability (days)) Number (double)
R^2	0.975
mean absolute error	10.164
mean squared error	358.158
root mean squared error	18.925
mean signed difference	-0.129
mean absolute percentage error	0.064
adjusted R^2	0.975

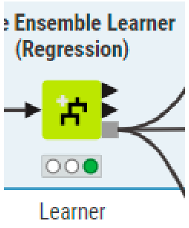
Ensemble Learner
(Regression)
Learner

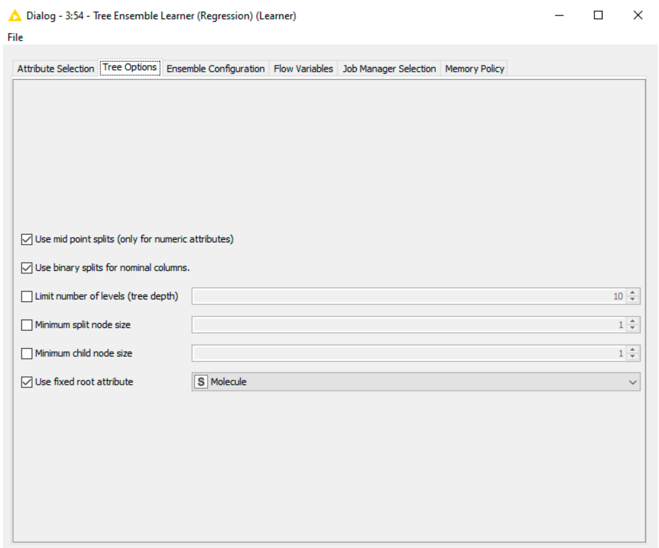
Dialog - 3:54 - Tree Ensemble Learner (Regression) (Learner)
File
Attribute Selection
Tree Options
Ensemble Configuration
Flow Variables
Job Manager Selection
Memory Policy
Use mid point splits (only for numeric attributes)
Use binary splits for nominal columns.
Limit number of levels (tree depth)
10
Minimum split node size
1
Minimum child node size
1
Use fixed root attribute
S Molecule

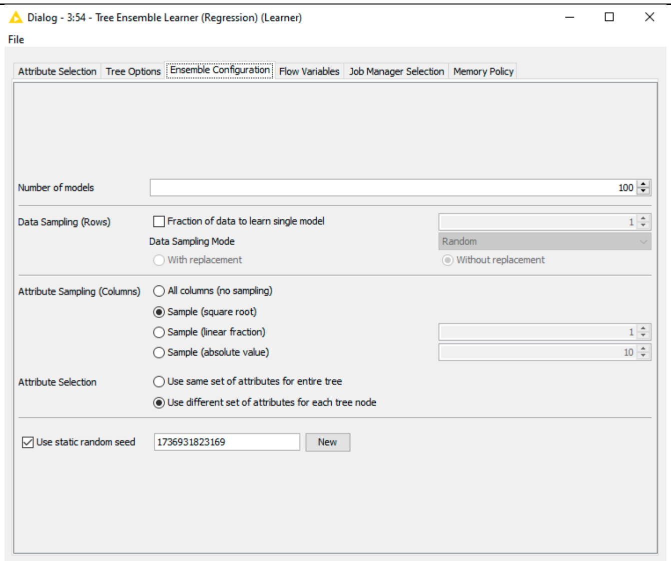
Dialog - 3:54 - Tree Ensemble Learner (Regression) (Learner)
File
Attribute Selection
Tree Options
Ensemble Configuration
Flow Variables
Job Manager Selection
Memory Policy
Number of models
100
Data Sampling (Rows)
Fraction of data to learn single model
1
Data Sampling Mode
Random
With replacement
Without replacement
Attribute Sampling (Columns)
All columns (no sampling)
Sample (square root)
Sample (linear fraction)
1
Sample (absolute value)
10
Attribute Selection
Use same set of attributes for entire tree
Use different set of attributes for each tree node
Use static random seed
1736931823169
New

<table>
<tr>
<td>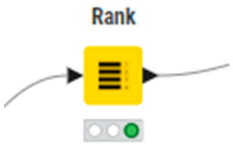
</td>
<td>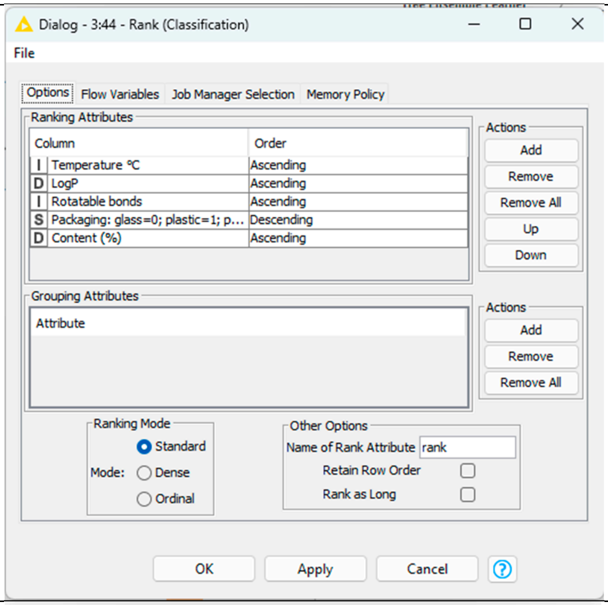
</td>
</tr>
<tr>
<td>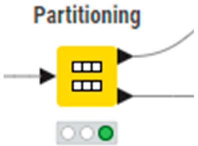
</td>
<td>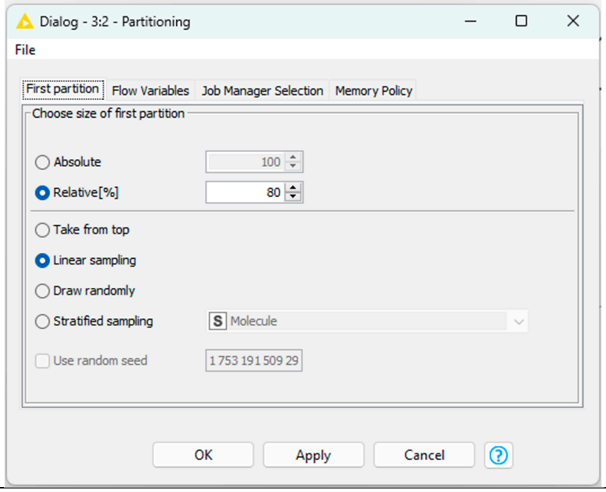
</td>
</tr>
</table>
